# Coping strategies in bipolar patients: A comparative study with siblings and healthy controls

**DOI:** 10.1192/j.eurpsy.2023.834

**Published:** 2023-07-19

**Authors:** M. Stambouli, B. N. Saguem, S. Bouhlel, I. Ben Mahmoud, W. Chebbi, J. Nakhli

**Affiliations:** psychiatry department, faraht hached hospital, sousse, Tunisia

## Abstract

**Introduction:**

Data regarding coping strategies used by bipolar patients to deal with psychosocial stress and their consequences in adaptational outcomes are scant. Moreover, family studies have reported the presence of several similarities between bipolar patients and their relatives regarding genetics, biology, personality traits, temperaments and stressful lived life experiences. Bipolar patients and their siblings had significantly higher global score of life events and more events in the field of work, socio-family events and health than control subjects. This might suggest that patients with bipolar disorder would be distinguished from their family members by the coping strategies they use to deal with stress.

**Objectives:**

In this study, we aimed to compare perceived stress and coping strategies of remitted bipolar I patients with those of their siblings and controls.

**Methods:**

A descriptive and comparative study of case-control type was conducted. Were included 46 bipolar I patients, 46 siblings and 50 controls. The three groups were matched for age and sex. Assessments of perceived stress and coping strategies were performed using the 10-item Perceived Stress Scale (PSS) and the 28-item Brief COPE respectively.

**Results:**

Mean age of bipolar I patients was 39 ± 13 years. Thirty-one patients (67%) reported family history of one or more psychiatric disorders.Mean duration of bipolar disorder was 11.83 ± 9.92 years.

There was no significant difference between the three groups on PSS scores. Bipolar patients and siblings were more likely to use emotion-focused coping strategies than controls (**p=0.001)**. Controls used problem-focused coping strategies more than bipolar patients (**p = 0.02)**. Compared to controls, bipolar patients were less likely to use active coping and planning, but they showed higher scores in the dimensions of humor, religion and behavioral disengagement with intergroup p value: **0.02; 0.019; 0.002** respectively.

**Conclusions:**

Our findings suggest that bipolar I patients were more likely to use maladaptive coping strategies to deal with stress than their siblings. Based on this observation, it seems advisable to study coping strategies used by bipolar patients, in order to reinforce adaptive strategies and to reduce maladaptive ones.

**Disclosure of Interest:**

None Declared

